# Degraded Carrageenan Causing Colitis in Rats Induces TNF Secretion and ICAM-1 Upregulation in Monocytes through NF-κB Activation

**DOI:** 10.1371/journal.pone.0008666

**Published:** 2010-01-13

**Authors:** Claudine Benard, Antonietta Cultrone, Catherine Michel, Carlos Rosales, Jean-Pierre Segain, Marc Lahaye, Jean-Paul Galmiche, Christine Cherbut, Hervé M. Blottière

**Affiliations:** 1 INRA, UR910 UEPSD, Domaine de Vilvert, Jouy-en-Josas, France; 2 INRA, UMR 1280 PHAN, Université de Nantes, CHU Hôtel-Dieu, Nantes, France; 3 INSERM, U913, CHU Hôtel-Dieu, Nantes, France; 4 INRA, BIA, Nantes, France; 5 Immunology Department, Instituto de Investigaciones Biomédicas, Universidad Nacional Autónoma de México, Mexico City, Mexico; Ohio State University, United States of America

## Abstract

Carrageenan (CGN) is a high molecular weight sulphated polysaccharide derived from red seaweeds. In rodents, its degraded forms (dCGN) can induce intestinal inflammation associated with macrophage recruitment and activation. The aim of this study was: 1) to analyze the size-dependent effects of dCGN on colon inflammation *in vivo*, and 2) to correlate these effects with monocyte/macrophage proliferation, cytokine production and expression of various cell surface antigens including ICAM-1 adhesion molecule. Peripheral blood monocytes (PBM) and THP-1 monocytic cells were cultured in the presence of either 10 or 40 kDa, dCGN. The 40 kDa, but not the 10 kDa dCGN, induced colitis in *in vivo*. Degraded CGN inhibited THP-1 cell proliferation *in vitro*, arresting the cells in G1 phase. In addition, dCGN increased ICAM-1 expression in both PBM and THP-1 cells with a major effect seen after 40 kDa dCGN exposure. Also, dCGN stimulated monocyte aggregation *in vitro* that was prevented by incubation with anti-ICAM-1 antibody. Finally, dCGN stimulated TNF-α expression and secretion by both PBM and THP-1 cells. All these effects were linked to NF-κB activation. These data strongly suggest that the degraded forms of CGN have a pronounced effect on monocytes, characteristic of an inflammatory phenotype.

## Introduction

Carrageenan (CGN) is a high molecular weight sulphated polysaccharide (>200 kDa) derived from red algae (*Rhodophyceae*). Three main forms of CGN have been identified: kappa, iota, and lambda. They differ from each other in sulphation degree and solubility [1], [2]. Native CGN is thought to be harmless and is widely used as a food additive to improve texture. It is also used in cosmetics and pharmaceuticals. However, acid treatment at high temperature (80°C) triggers CGN hydrolysis to lower molecular weight (<50 kDa) compounds known as poligeenan or degraded CGN (dCGN). These dCGNs induce inflammation and have been widely used as models of colitis in several species, including rats [3], rabbits [4] and guinea pigs [5]. The role of dCGN as a tumor-promoting factor remains controversial [4], [6]–[8].

Although the native form is thought to be harmless for human consumption, small amounts of dCGN are probably produced by acid hydrolysis during gastric digestion [9], [10] or interaction with intestinal bacteria [11], [12]. Whereas the effects of native and dCGN on intestinal inflammation have been extensively analyzed in animal models, only few studies have been conducted using human cell lines. Recent studies have shown a link between exposure to native form CGN and IL-8 production by the human intestinal epithelial cell line, NCM460, *via* Nuclear Factor-κB (NF-κB) activation [13], [14]. NF-κB is a transcription factor that regulates the expression of genes associated with inflammation [15], [16].

Macrophage infiltration and accumulation is a common characteristic of intestinal diseases [17]. Macrophages represent 10% of total *lamina propria* cells, secrete a wide range of biologically active compounds and express cell-adhesion molecules. The immune cell response to an inflammatory stimulus seems to be amplified or directly generated by cells exposed to sulphated polysaccharides such as carrageenans. Indeed, inflammation induced by dCGN was associated with recruitment of macrophages to inflammation sites [18], [19]. Also, inflammation induced by Dextran Sulphate Sodium (DSS), another sulphated compound, was directly associated with macrophages recruitment [20], since DSS still provoked inflammation after T-lymphocyte and NK cell depletion [20]. Although inflammation can be induced by dCGN, there are no data on human monocyte responses to dCGN exposure. Therefore, to investigate the effects of dCGN on human monocytes, normal Peripheral Blood Monocytes (PBM) and tumoral monocyte/macrophage THP-1 cells were exposed to 10 kDa and 40 kDa dCGN. We found that dCGN inhibited THP-1 cell proliferation *in vitro*, increased ICAM-1 expression, stimulated ICAM-1-dependent monocyte aggregation, and stimulated TNF-α expression and secretion. These responses were more pronounced after 40 kDa dCGN exposure and were linked to NF-κB activation. In addition, the 40 kDa dCGN, but not the 10 kDa dCGN induced *in vivo* colitis as shown by the inflammatory response in the rat colon. These results suggest that the degraded forms of CGN have an important effect on monocytes resulting in an inflammatory phenotype.

## Materials and Methods

### Preparation of Degraded Carrageenan

Two preparations of degraded carrageenan with low, (∼10 kDa; C10), and medium, (∼40 kDa; C40) molecular weight were prepared from native iota-carrageenan extracted from *Euchema spinosum* (generously provided by Sanofi Biosystems Industry, Boulogne-Billancourt, France). Native carrageenan was dissolved in distilled water (5% w/v) under vigorous stirring and heated to 60°C. Then, the carrageenan solution was submitted to two different treatments to obtain both low and medium molecular weight fractions. Briefly, for the low molecular weight fraction, carrageenan solution was hydrolyzed with 0.3% (v/v) concentrated sulphuric acid for 15 min at 80°C. After neutralization with NaOH 4N, the solution was ultra filtered through a hollow fibre cartridge with MW cut-off 5 kDa, (Amicon Inc, Beverly, USA). For the medium molecular weight fraction, the carrageenan solution was hydrolyzed with 0.3% (v/v) concentrated sulphuric acid for 30 min at 60°C. After neutralization, the supernatant was ultra filtered (MW cut-off 100 kDa). The filtrate was submitted to a second ultra filtration (MW cut-off 5 kDa). Both preparations of dCGN were precipitated with 4 volumes of 95% ethanol, dried at room temperature and ground to small particles (1 mm in diameter). Using gel-permeation chromatography in combination with light scattering measurements (see Viebke et al. [21]), it was confirmed that the low fraction had an average molecular weight of 10 kDa, and the medium fraction of 40 kDa. The sulphate content of polysaccharides in both fractions was measured following the method of Quemener et al. [22]. Finally, the absence of polysaccharide structure modifications in the two fractions was confirmed using ^2^H-NMR spectroscopy. The absence of LPS contamination in the two fractions was confirmed using the e-Toxate^®^ kit (Sigma, St Quentin Fallavier, France). Before use in cell culture, the two fractions were dissolved in complete medium during 30 min at 56°C.

### Animals, Chemicals and Diet

Male Wistar rats (150 g average weight) were housed under standard conditions and fed *ad libitum* with standard rodent laboratory chow. Degraded iota-carrageenans were administered in the drinking water (5% w/v) for 55 days to 2 groups of six animals each. The first group received the low molecular weight carrageenan (10 kDa dCGN) and the second received the medium molecular weight carrageenan (40 kDa dCGN). An additional group of four rats were maintained on regular tap water (control group). To increase palatability 0.2% sucrose was added to the drinking water of all groups (Van der Waaji et al., [23]). Fresh carrageenan solutions were prepared daily.

### Evaluation of Colitis

Body weight, liquid and food consumption, diarrhea and rectal bleeding (detected by eye inspection) were recorded throughout the feeding period. After 55 days, animals were sacrificed by cervical dislocation. The length of the colon was measured as described by Okayashu et al. [24]. Then, each colon was ligated in sections of 2 cm and 1 to 2 ml of 10% formalin was infused into the intestinal lumen. The moderately distended segment was sectioned and fixed in 10% formalin. The following day, the intestinal content was removed by vortexing. The fixed segment was kept in 10% formalin at 4°C until the paraffin embedding procedure. To evaluate the degree of inflammation, this segment of colon was opened longitudinally and macroscopic and histological scores of inflammation were recorded as previously described [25], [26]. The toluidine blue staining was used for identification of sulphated polysaccharides in the intestinal mucosa. On the day of sacrifice, a fresh sample of each colon (50 mg) was collected for myeloperoxidase (MPO) assay according to Krawisz et al., [27]. The level of MPO, mainly expressed by neutrophils, indicates the rate of recruitment of neutrophils to the intestinal mucosa. One unit of MPO activity corresponds to the degradation of 1 µmol of peroxide per minute at 25°C.

### Cell Culture

All tissue culture reagents were from Invitrogen (Cergy Pontoise, France). THP-1 human monocytic cells were maintained in RPMI-1640 supplemented with 10% FCS, 2 mM L -glutamine, 50 U/ml penicillin and 50 mg/ml streptomycin at 37°C in a 5% CO_2_ incubator. Human peripheral blood mononuclear cells were obtained from heparinized blood by Ficoll-Hypaque density gradient. Monocytes were then isolated by adherence to culture flasks as described [28]. For cell aggregation, monocytes were cultured in the presence or absence of C10 or C40 for 72 h. Cell colonies were monitored under an inverted phase contrast microscope coupled through a video camera to a computer. In some wells, neutralizing monoclonal antibody to ICAM-1 (2.5 µg/ml) (Tebu, Le Perray en Yvelines, France) was added.

### Cell Cycle Analysis

THP-1 cells in exponential growth phase were exposed to complete medium in the presence or absence of carrageenans for 24 h before being stained with propidium iodide using the DNA-Prep Coulter kit according to the manufacturer's instruction (Beckman-Coulter, Villepinte, France). Cell DNA content was then analyzed by flow cytometry using an EPICS XL2 (Beckman-Coulter). Raw data for the distribution of DNA content of 30,000 cells retrieved from the cytometer were expressed as the percentage of G0/G1 through G2/M populations. Multicycle AV software (Phoenix Flow Systems, San Diego, CA) was used to generate DNA content frequency histograms and facilitate data analysis.

### Cell Surface Antigen Expression Analysis

Peripheral Blood Monocytes or THP-1 cells were exposed to complete medium in the presence or absence of carrageenan for 36 h. After two washes in PBS without Ca^2+^ and Mg^2+^, cells were incubated in PBS containing 0.1% gelatin and 8% AB human serum to prevent binding to Fc receptors. Then, 5×10^5^ cells were incubated with primary antibodies at 4°C for 30 min. Two other washes in PBS preceded incubation with FITC-conjugated goat antibody anti-mouse IgG diluted 1/1000 at 4°C for 30 min (Tebu). After two additional washes, analysis of stained cells was performed on an EPICS XL2 (Beckman-Coulter). The cell population was gated according to its forward and wide-angle light scattering. Data were expressed as mean relative fluorescence intensity (MFI) of 3000 cells.

### TNF Activity Bioassay

Monocytes or THP-1 cells were cultured with or without different concentrations of CGNs or LPS (*Salmonella typhosa*, Sigma) for 24 h or the indicated time. Biologically active TNF-α/β in tissue culture supernatant was measured using the WEHI 164 clone 13-cell killing assay [29]. TNF concentrations are expressed as pg/ml.

### RT-PCR Analysis

Total RNA from monocytes was isolated using TRIzol Reagent™ (Invitrogen). cDNA was generated on 1 µg of total RNA in a reaction volume of 20 µl, using M-MLV reverse transcriptase (Invitrogen). PCR was done in the linear range of amplification (determined for each primer pair-cDNA combination). Standard PCR reactions were performed with 1 µl of the cDNA solution, 50 µM of each primer solution, 10 mM of each dNTP, 25 mM MgCl_2_, 10X Goldstar DNA polymerase reaction buffer, and 0.5 units of Goldstar DNA polymerase (Eurogentec, Seraing, Belgium). First PCR cycle consisted of 1 min at 92°C, 1 min at 58°C and 1 min at 72°C; then each PCR cycle consisted of 40 sec at 92°C, 40 sec at 58°C and 50 sec at 72°C. cDNA for β-actin was amplified for 28 cycles using the oligos: sense 5′-GGCATCGTGATGGACTCCG-3′ and antisense 5′GCTGGAAGGTGGACAGCGA-3′. cDNA for TNF-α was amplified for 35 cycles using the oligos: sense 5′-AAGCCTGTAGCCCATGTTGT-3′ and antisense 5′-CAGATAGATGGGCTCATACC-3′. cDNA for ICAM-1 was amplified for 35 cycles using the oligos sense 5′-GTAGCAGCCGCAGTCATAATGG-3′ and antisense 5′-A TGCTGTTGTATCTGACTGAGG-3′.

### NF-kB Transcription Reporter Gene Assay

The plasmid 3XMHC-luc (a generous gift from Drs. J. Westwick and D.A. Brenner, University of North Carolina, Chapel Hill) contains three copies of NF-κB-responsive element from the MHC class I locus, placed upstream of the luciferase gene. Human monocytic THP-1 cells were transiently transfected as previously described [30], and then cultured for 4 h alone or with increasing concentration of either C10 or C40. Luciferase activity was determined using a luminometer (Monolight 2010 Luminometer, Ann Arbor, MI).

### Western Blot Analysis

THP-1 cells were stimulated for various lengths of time with 0.1 mg/ml C10 or C40, or 10 µg/ml LPS. Cells were then pelleted, washed and homogenised in lysis buffer (10 mM Hepes, pH 7.9, 150 mM NaCl, 1 mM EDTA, 0.6% NP-40, and 0.5 mM PMSF) on ice. Homogenates were sonicated, centrifuged at 10,000 rpm to remove cellular debris, and supernatant collected. Protein concentration was determined using the *DC* Protein Assay (Bio-Rad). Proteins in samples (15 µg total proteins) were resolved in a denaturing 12% polyacrylamide gel and transferred to a nitrocellulose membrane. I-κBα protein was detected using a rabbit polyclonal antibody (Santa Cruz Biotechnology, CA) followed by a horseradish peroxidase-coupled goat polyclonal antibody against rabbit Ig (Caltag Laboratories). Finally, IκB bands were revealed using the ECL™ detection system (Amersham Pharmacia Biotech, Les Ullis, France) according to the manufacturers' instruction. Antibody to α-Tubulin (Santa Cruz) was use as loading control.

For nuclear NF-κB, THP-1 cells were stimulated with 1 mg/ml C10 or C40 for 30 minutes at 37°C. Cells were then pelleted and nuclei separated as described [31]. Nuclei were washed and homogenized directly in loading (Laemli) buffer and heated for 5 minutes at 100°C. Proteins in samples were resolved in a denaturing 8% polyacrylamide gel and transferred to a polyvinylidine fluoride (PVDF) membrane (Immobilon-P; Millipore, Bedford, MA). Membranes were incubated in blocking buffer (1% BSA, in PBS) for two hours at room temperature. Membranes were subsequently probed with the corresponding antibody in blocking buffer, overnight. Rabbit polyclonal antibody anti-NF-κB p50 subunit (# sc-114) or anti-NF-κB p65 subunit (# sc-109) from Santa Cruz Biotechnology were used. Membranes were washed six times in PBS with 0.05% Tween 20, 5 minutes each time, and incubated with a 1/3000 dilution of HRP-conjugated F(ab')2 goat anti-rabbit IgG in 5% nonfat dry milk and 0.05% Tween 20 in PBS for 1 hour at room temperature. After washing six more times in PBS with 0.05% Tween 20, antibody-reactive proteins were detected using a chemiluminescence substrate (SuperSignal; Pierce, Rockford, IL) according to the manufacturer's instructions. To confirm that equivalent amounts of protein were loaded in each line, membranes were also Western blotted for ERK as described [32].

### Analysis of NF-κB Activation by Flow Cytometry

Nuclear activation of NF−κ*Β* by flow cytometry was performed as described [31].

### Statistical Analysis

The results were expressed as the mean value ± S.E.M. of individual experiments. The statistical significance of the differences between mean values was assessed by the Student's t-test and analysis of variance (ANOVA).

## Results

### Degraded CGN Induce Colonic Inflammation

**Figure 1:**
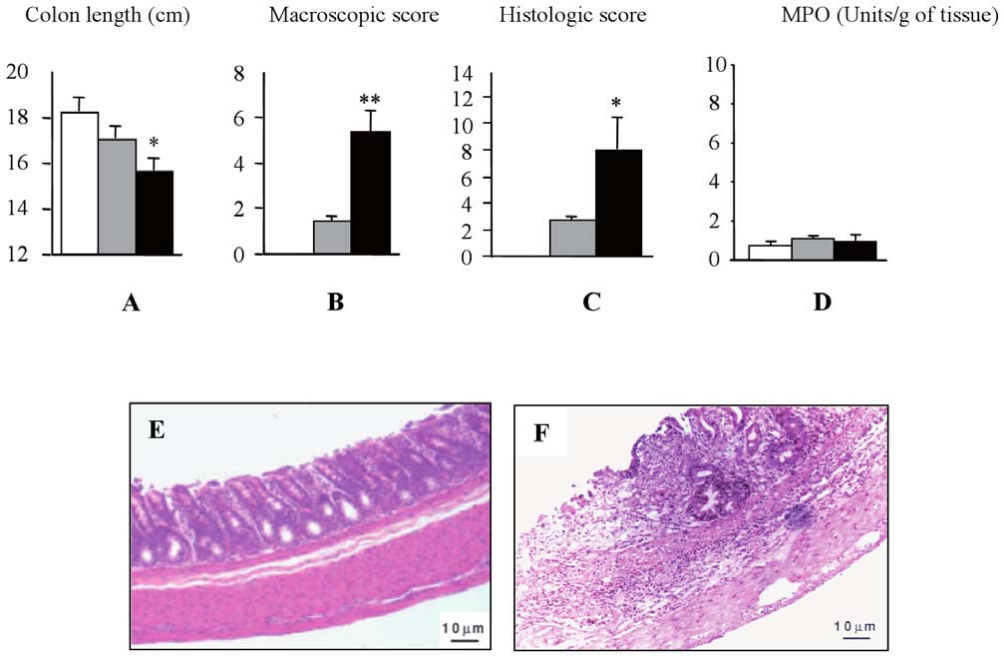
Degraded CGN induced colon inflammation in rats. Histograms showing the effect of degraded CGN on: colon length (**A**); macroscopic (**B**) and histological (**C**) inflammation score of colon; Myeloperoxidase (MPO) activity (**D**). Control rats (white bars); 10 kDa degraded CGN-treated rats (grey bars); 40 kDa degraded CGN-treated rats (black bars). * p<0.05 from control. ** p<0.01 from control. Histological analysis of colon from control rats (**E**), and from 40 kDa dCGN-treated rats (**F**).

All rats developed diarrhea during degraded carrageenan administration and gross evidence of blood was frequently detected in the stools. Colon length dramatically decreased in all treated rats with a more pronounced effect being observed in the 40 kDa dCGN treated group (Fig. 1A). Furthermore, prolonged exposure to 40 kDa dCGN resulted in high macroscopic and histological scores of inflammation (Fig. 1B, C). Only weak myeloperoxidase activity was detected in both control and dCGN-treated groups (Fig. 1D), indicating that granulocytes did not play a major role in the inflammation at that stage. Histological examination revealed various degrees of mucosal inflammation. Rats treated with 10 kDa dCGN showed edema, epithelium atrophy and slight lymphocyte infiltration (data not shown). These symptoms were totally absent in the colon of control rats (Fig. 1E). More severe mucosal injuries including ulceration, hyperplastic epithelium, crypt distortion and a strong macrophage infiltration, were observed in the 40 kDa dCGN-treated rats (Fig. 1F). No sulphated polysaccharides were detected by toluidine blue staining of colon mucosa from rats treated with either the 10 or 40 kDa dCGN (not shown). Although we cannot exclude that dCGN mat not have retained in the section during the histology procedure, this indicates that these polymers may not have been phagocytosed.

### Degraded CGN Induced-TNF-α Production by Monocytes *In Vitro*

**Figure 2:**
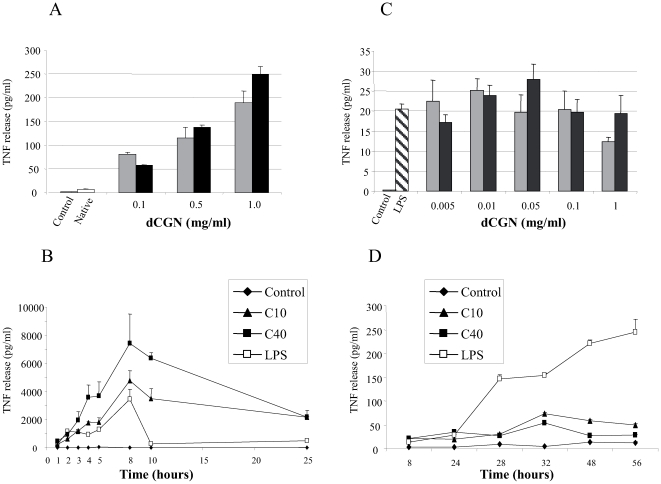
Degraded CGN stimulated TNF secretion from monocytes. Levels of TNF released from peripheral blood monocytes (**A–B**) and THP-1 cells (**C–D**) after stimulation with dCGN. **A**: TNF release induced by native CGN (open bars), 10 kDa dCGN (grey bars), or 40 kDa dCGN (black bars). **B**: Kinetics of TNF release induced by nothing (control; black diamonds), 0.1 mg/ml 10 kDa dCGN (black triangles), 0.1 mg/ml 40 kDa dCGN (black squares), or 10 µg/ml LPS (open squares). **C**: TNF release induced by nothing (control; open bars), 10 µg/ml LPS (hatched bars), or increasing concentrations of 10 kDa dCGN (grey bars), or 40 kDa dCGN (black bars). **D**: Kinetics of TNF release induced by nothing (control; black diamonds), 0.1 mg/ml 10 kDa dCGN (black triangles), 0.1 mg/ml 40 kDa dCGN (black squares), or 10 µg/ml LPS (open squares).

In order to study the capacity of dCGN to stimulate TNF-α production, peripheral blood monocytes were cultivated in the presence of dCGN (0.1 to 1 mg/ml). Very low levels of TNF-α were induced in PBM after stimulation with native CGN (Fig. 2A). Addition of 0.1 mg/ml 10 kDa dCGN resulted in approximately a 60-fold increase in TNF-α production by PBM. This was a dose-dependent effect that reached a 180-fold increase when cells were exposed to 1 mg/ml of 10 kDa dCGN (Fig. 2A). A 250-fold increase in TNF-α production was detected at 1 mg/ml 40 kDa dCGN (Fig. 2A). TNF-α production increased in time reaching a maximum level at 8 hours of culture (Figure 2B). After 24 h, the amount of secreted TNF-α was still one third of the total TNF-α. Lipopolysaccharide (LPS), a known activator of immune cells also induced TNF-α production with similar kinetics as dCGN (Fig. 2B). However, the amount of TNF-α produced by LPS was 4-fold less than the one produced by dCGN and it was not detected after 8 hours of culture (Fig. 2B). Similarly, monocytic THP-1 cells cultivated in the presence of variable concentration of dCGN showed an increase in TNF-α production (Fig. 2C). This increase in TNF-α production was significantly smaller (about 10-fold) than the one presented by PBM (Fig. 2A). No TNF-α was released from THP-1 cells exposed to native CGN (not shown). TNF-α production by THP-1 cells was not dose dependent to the amount of dCGN used. Also there was no difference between the two forms (10 and 40 kDa) of dCGN (Fig. 2C). Interestingly, TNF-α release from THP-1 cells stimulated with dCGN reached a maximum level at 32 h, while stimulation with LPS reached a maximum level at 56 h (Fig. 2D).

### Effect of Native and Degraded CGN on THP-1 Proliferation and Cell Cycle

**Figure 3:**
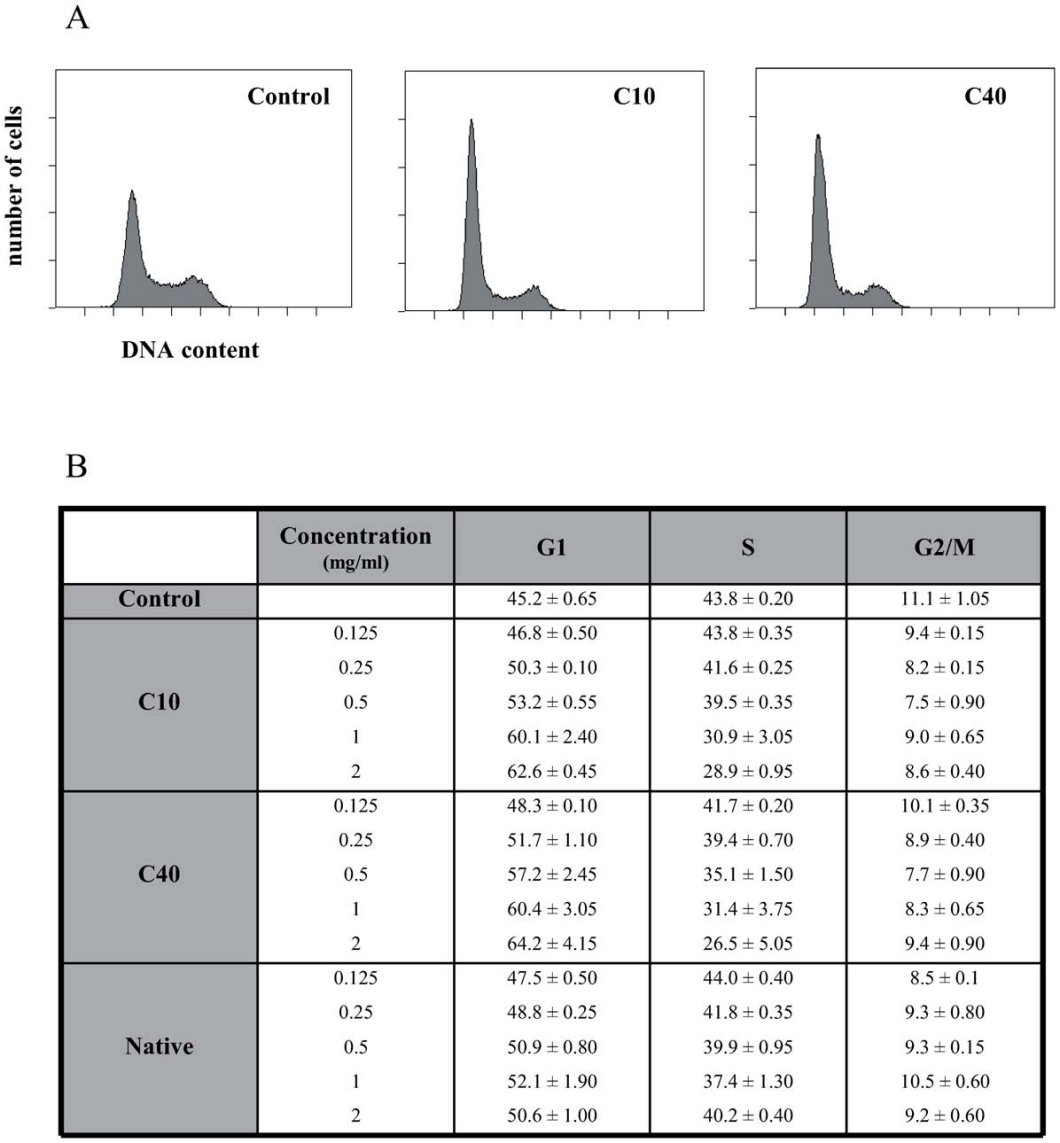
Degraded CGN induced THP1 cell cycle arrest in G1 phase. THP-1 cells in exponential growth phase were incubated in the presence or absence of carrageenan for 24 h before being stained with propidium iodide. Cell DNA content was then analyzed by flow cytometry. **A:** Histograms of cells treated with medium only (control), 10 kDa dCGN (C10), or 40 kDa dCGN (C40). **B:** Percentage of cells in each phase of the cell cycle when treated with medium only (control), different concentrations of 10 kDa dCGN (C10), of 40 kDa dCGN (C40), or of native CGN (Native).

Preliminary observations by enumeration of THP-1 cells exposed to different concentrations of native and dCGN (10 and 40 kDa) during 2, 5 and 7 days, showed a decline in cell number (data not shown). This suggested that dCGN might cause an alteration in the cell cycle. Cell cycle analysis using flow cytometry showed an accumulation of THP-1 cells in G0/G1 phase, which was associated with a decrease number of cells in the S phase (Fig. 3A). The percentage of cells in the G0/G1 phase was 45.2% for control cells, 62.6% for C10 dCGN (at 2 mg/ml), and 64.2% for C40 dCGN-treated cells (at 2 mg/ml) (Fig. 3B). The effect of dCGN on cell cycle was dose-dependent (Fig. 3B). Neither native nor dCGN had an effect on the number of cells in the G2/M phase (Fig. 3). This effect is not due to cytotoxicity of dCGN even at the highest concentration (i.e. 2 mg/ml) since cell viability was not affected (data not shown).

### ICAM-1 Expression Is Induced by Degraded CGN and Is Responsible for Monocytes Aggregation *In Vitro*

**Figure 4:**
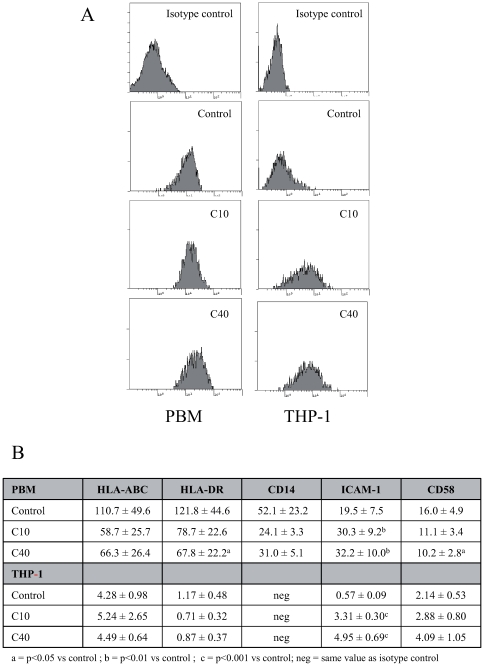
Degraded CGN stimulated ICAM-1 expression in monocytes. Peripheral blood monocytes (PBM) or THP-1 cells were incubated in the presence or absence of carrageenan for 24 h before being stained for various cell surface antigens. Antigen expression was then analyzed by flow cytometry. **A**: Histograms of ICAM-1 expression in cells treated with medium only (control), 10 kDa dCGN (C10), or 40 kDa dCGN (C40). **B**: Fluorescence intensity for expression of the antigens HLA-ABC, HLA-DR, CD14, ICAM-1, and CD58 in cells treated with medium only (control), 10 kDa dCGN (C10), or 40 kDa dCGN (C40). Data are mean +/− SEM.

In order to study the effect of dCGN on the expression of cell surface antigens, PBM and THP-1 cells were incubated for 36 h in the presence and absence of dCGN. The expression of various cell surface molecules was analyzed by flow cytometry as described in materials and methods. Both forms of dCGN clearly stimulated expression of ICAM-1 (CD54) on PBM and THP-1 cells (Fig. 4A). The increase in ICAM-1 expression was higher on THP-1 cells treated with 40 kDa dCGN (Fig. 4B). Another surface antigen, the lymphocyte function-associated antigen 3 (CD58) was slightly reduced on PBM after treatment with 40 kDa dCGN (Fig. 4B). Interestingly, expression of major histocompatibility complex molecules of class I (HLA-ABC) and of class II (HLA-DR), as well as the monocyte marker CD14, seemed to be reduced by treatment with dCGN (Fig. 4B). However, these differences were not statistically significant.

**Figure 5:**
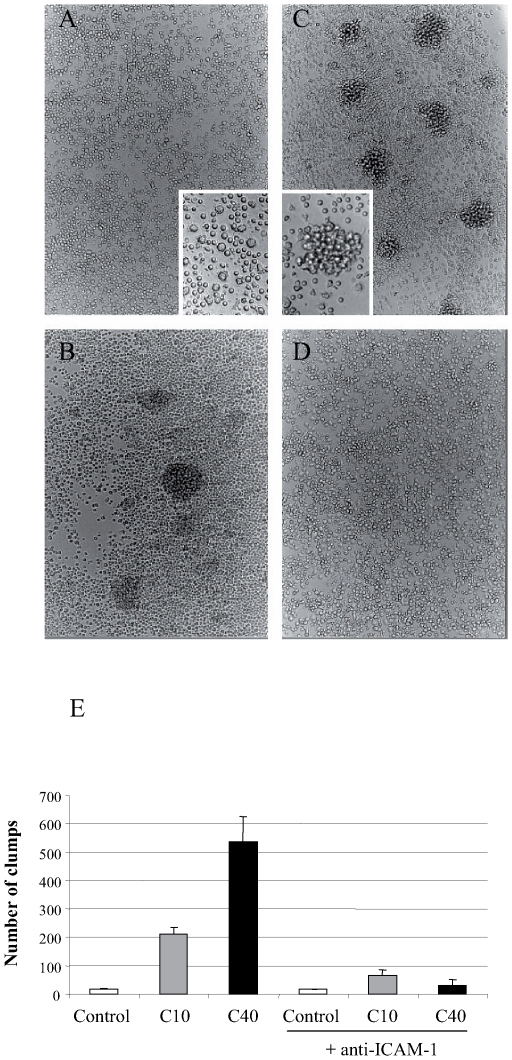
Degraded CGN induced monocytes aggregation *in vitro*. Monocytes were incubated in the absence (**A**) or the presence of 1 mg/ml 10 kDa dCGN (**B**), 1 mg/ml 40 kDa dCGN (**C**), or 1 mg/ml 40 kDa dCGN plus 2.5 µg/ml anti-ICAM-1 antibody (**D**). Cells were observed by phase contrast inverse microscopy at 150X magnification. Inserts in A and B show a close up of cells at 300X magnification. **E**: Number of monocyte aggregates in 24 wells plate of PBM cell cultured with nothing (control), with 10 kDa degraded CGN (C10), or with 40 kDa degraded CGN (C40). Some cultures had also 2.5 µg/ml anti-ICAM-1 antibody. Data are mean +/− SEM.

Treatment with dCGN also induced a strong aggregation of monocytes, detected by phase contrast inverse microscopy (Fig. 5). Although this effect was easily observed in monocytes incubated with the 10 kDa dCGN (Fig. 5B), a more robust cell aggregation was observed in monocytes incubated with the 40 kDa dCGN (Fig. 5C). ICAM-1 has been proposed to be the main adhesion molecule responsible for monocyte aggregation. To confirm this, monocytes were incubated with both types of dCGN in the presence of an anti-ICAM-1 antibody, an anti-CD58 antibody and an isotype control IgG1 antibody. The anti-ICAM-1 antibody effectively blocked the cell aggregates induced by dCGN (Fig. 5D, 5E), strongly suggesting that indeed ICAM-1 is responsible for monocyte aggregation. Both the control IgG1 and the anti-CD58 antibody did not modify monocyte aggregation (data not shown).

### Degraded CGN Induce an Increase in ICAM-1 and TNF-α mRNA Expression

**Figure 6:**
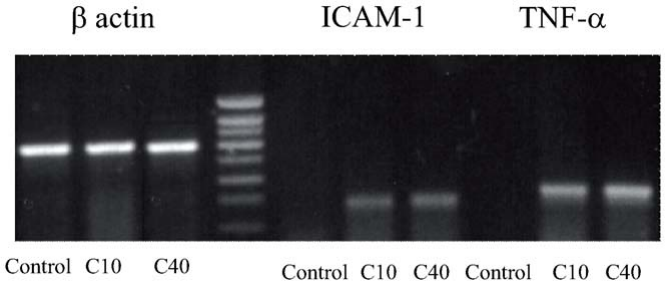
Degraded CGN stimulated ICAM-1 and TNF-α gene expression in monocytes. Representative samples of RT-PCR analysis showing over expression of ICAM-1 and TNF-α after stimulation of monocytes with 1 g/l of degraded CGN. β-actin expression was used as normalization gene.

The increase in surface ICAM-1 expression and TNF-α production by monocytes correlated with an upregulation of mRNA for these molecules. Both 10 kDa and 40 kDa dCGN induced a robust increase in mRNA for both ICAM-1 and TNF-α (Fig. 6). β-actin mRNA levels were not affected by dCGN treatment.

### Degraded CGN Induce IκB Degradation and NF-κB Activation

**Figure 7:**
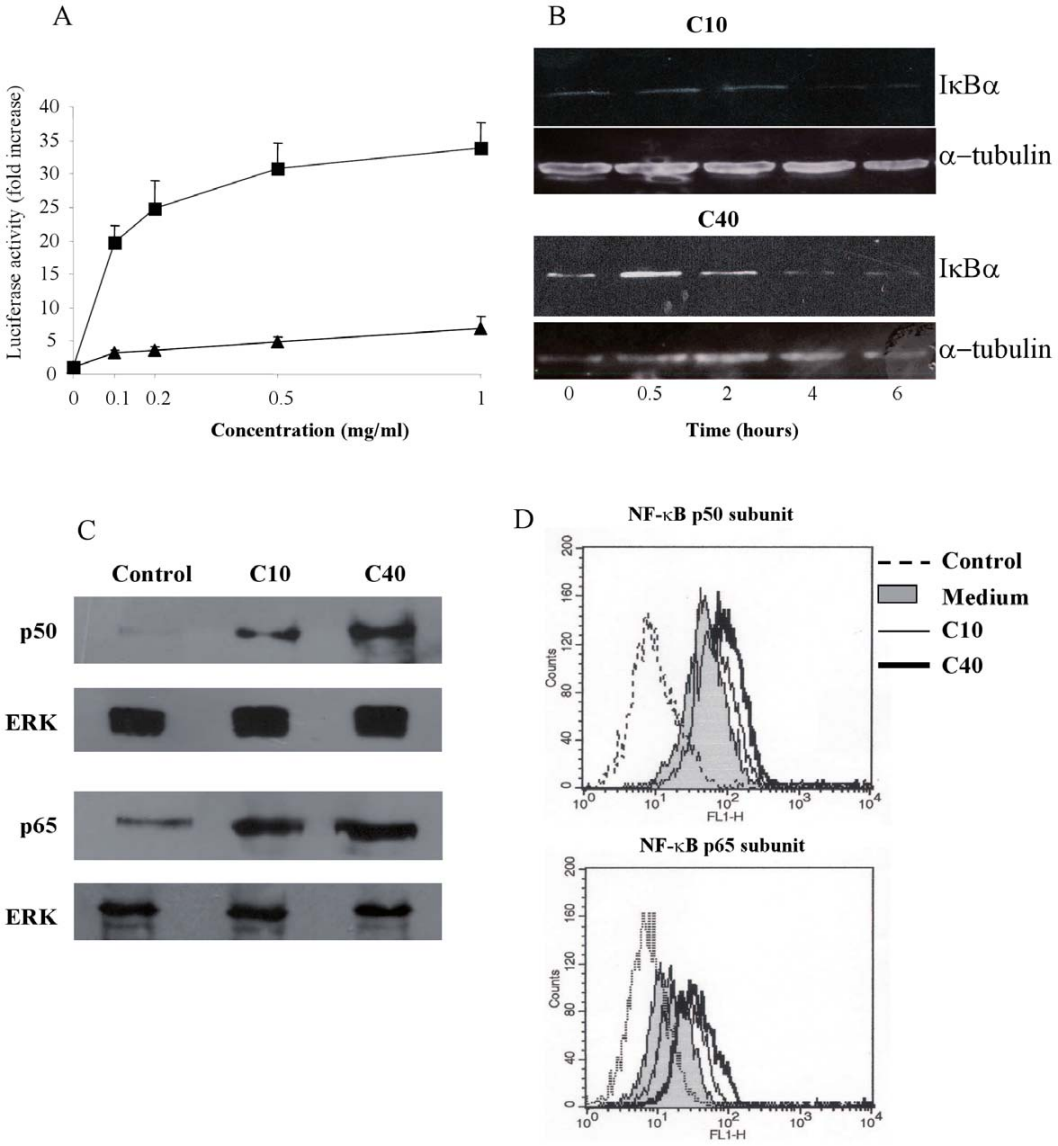
Degraded CGN activated the NF-kB pathway in monocytes. **A**: THP-1 cells were transfected with a NF-κB reporter plasmid driving expression of luciferase. Cells were then treated with various concentrations of 10 kDa (triangles), or 40 kDa dCGN (squares). **B**: THP-1 cells treated with 1 mg/ml of 10 kDa dCGN (C10), or with 1 mg/ml of 40 kDa dCGN (C40) were lysed after various periods of time. Proteins in cell extracts were resolved by SDS-PAGE and then Western blotted for IκBα or α−tubulin as loading control. **C**: Degraded carrageenans (dCGN) induced activation of NF-κB. THP-1 cells were treated with nothing (control), or with 1 mg/ml of 10 kDa dCGN (C10), or with 1 mg/ml of 40 kDa dCGN (C40) for 30 minutes at 37°C. Nuclei were isolated and lysed. Proteins in nuclear extracts were resolved by SDS-PAGE and then Western blotted for NF-κB p50 subunit (p50) or NF-κB p65 subunit (p65). Lower panels show Western blots of nuclear ERK revealing equivalent amount of protein in each sample. Data are representative of three separate experiments. **D**: Degraded carrageenan (dCGN) induced activation of NF-κB. Nuclei isolated from THP-1 cells were fluorescence-stained for NF-κB p50 subunit or NF-κB p65 subunit before (filled area) or after cells were treated with 1 mg/ml of 10 kDa dCGN (C10), or with 1 mg/ml of 40 kDa dCGN (C40) for 30 minutes at 37°C. Dashed line corresponds to nuclei stained only with secondary fluorescence antibody. Fluorescence intensity was analyzed by flow cytometry as described.

The expression of genes encoding for ICAM-1 and TNF-α is controlled by the nuclear factor NF-κB. Site-specific phosphorylation of the inhibitor IκB leads to its degradation by proteasome and to a consequential activation of the NF-κB pathway. Using a reporter plasmid for NF-κB activation, it was confirmed that dCGN induced a strong activation of NF-κB, as reflected by an increase in luciferase activity (Fig. 7A). Both forms of dCGN used induced NF-κB activation in a dose dependent manner. However, the effect was more strongly induced by the 40 kDa dCGN (Fig. 7A). These results were further confirmed by directly detecting NF-κB in the cell nucleus by Western blotting (Fig. 7C) and by FACS (Fig. 7D). These assays also allowed us to determine what NF-κB subunits were activated by dCGN. Both forms (10 or 40 kDa) of dCGN induced activation of the p50 and p65 subunits of NF-κB. This nuclear factor was present in low levels in the cell nucleus and increased considerably after treatment with dCGN. Western blots suggested the the 40 kDa form of dCGN induced a stronger activation of NF-κB (Fig. 7C). A more sentive assay for nuclear factor activation is flow cytometry of nuclei stained with specific antibodies for the nuclear factor of interest. In agreement with the previous data, FACS analysis of nuclei from THP-1 cells showed that there was a basal level of nuclear NF-κB (Fig. 7D). Again, both forms (10 or 40 kDa) of dCGN induced an increase of the p50 and p65 subunits of NF-κB in the nucleus of these cells. The 40 kDa degraded CGN gave a stronger increase of NF-κB (Fig. 7D). These data strongly suggest that the heterodimer p50/p65 is the NF-κB isoform activated by degraded CGN in monocytes. In addition, degradation of the inhibitor IκBα was also observed in cells treated with dCGN (Fig. 7B). No significant IκBα degradation was detected within two hours of dCGN treatment, but IκBα was markedly degraded by four hours of dCGN treatment (Fig. 7B). We focused on IκBα subunit, since it masks the nuclear localisation sequence of p65, it is the most rapidly degraded subunit and the most studied one.

## Discussion

Inflammation of the intestinal tract is usually associated with infiltration and activation of intestinal macrophages [17]. These macrophages are able to initiate immune responses and can be induced to differentiate into cells that either exacerbate or inhibit the inflammation. Accumulation of different types of leukocytes, including monocytes/macrophages, neutrophils, and lymphocytes in the intestinal mucosa during inflammation, is normally followed by secretion of pro-inflammatory cytokines [33]. Several stimuli can induce these leukocytes to produce and secrete cytokines during inflammation. One of the most potent and known stimuli for leukocyte activation is LPS from Gram-negative bacteria [34]. In addition, other factors are also able to stimulate cytokine secretion from various leukocytes [35], [36]. One such factor is CGN, a high molecular weight sulphated polysaccharide (>200 kDa) derived from red algae (*Rhodophyceae*) [1], [2]. Native CGN is widely used as a food additive (E 407) to improve texture. It is also used in cosmetics and pharmaceuticals. Although native form CGN (200–800 kDa) has been declared harmless to humans [8], its degraded forms (<50 kDa), also known as poligeenan, are widely used to induce colitis in rodents [3]–[5]. These degraded CGN may also have a possible carcinogenic effect [4], [6]–[8]; however this is still controversial.

Although acid treatment at high temperature (80°C) is required for CGN hydrolysis *in vitro* to lower molecular weight dCGN, it is probable that some dCGN are produced by acid hydrolysis during gastric digestion [9], [10] or interaction with intestinal bacteria [11], [12]. Thus, understanding the mechanisms of dCGN-induced bowel inflammation is of great importance. In this report, we have analyzed the role of human monocytes (PBM and THP-1) in dCGN-induced inflammation.

Preliminary *in vivo* studies in rats treated with dCGN revealed significant shortening of the large intestine associated with an inflammatory state, *i.e.* strong infiltration of macrophages to the intestinal mucosa similar to DSS-induced inflammation [20]. Using two fractions of dCGN (10 and 40 kDa), we observed a strong correlation between the severity of the inflammation and the dCGN molecular size, thus confirming the size related inflammation *in vivo*. This macrophage accumulation was not due to cell proliferation because dCGN inhibited THP-1 monocytes proliferation *in vitro*. These results are similar to those obtained with human colonic epithelial cells (NCM460 cell line) exposed to native CGN for 1–8 days [37]. Thus, it seems that dCGN promote macrophage infiltration by recruiting new cells to the inflamed intestinal mucosa and not by inducing cell proliferation. In addition, very few polymorphonuclear cells were detected in the mucosa at the time point analyzed as demonstrated by a very low level of MPO in the intestinal tissue.

These results suggest that monocytes might produce cytokines associated with activation into macrophages in response to dCGN. Thus, we analyzed the production of TNF by both PBM and THP-1 cells in response to dCGN. Degraded CGN induced a robust production of TNF by monocytes. The 40 kDa form of dCGN was more potent for monocyte stimulation than the 10 kDa or the native ones. Surprisingly, monocyte activation by dCGN to produce TNF was much stronger than the activation induced by LPS, an inflammatory factor considered to be among the most potent stimuli for leukocyte activation. These results underline the fact that partially degraded forms of CGN have important cellular effects. The amount of TNF secreted by PBM induced with LPS was much larger than the one secreted by THP-1 monocytes. Since monocyte activation by LPS is associated with the presence of the CD14 and TLR4 receptors [38]–[40], the different response observed could be due to the different expression of these receptors in PBM and THP-1 cells. CD14 is not expressed by THP-1 monocytes, but it is expressed by PBM. This fact could explain why THP-1 cells produced a much smaller response to LPS than PBM and also the difference of kinetics in the LPS induced TNF secretion. The peak of TNF response was observed after 8 h stimulation followed by a rapid decrease to baseline at 10 h, whereas on THP-1 cells the peak was not reached until after 56 h. Moreover, the amount of TNF secreted by monocytes induced with dCGN was much larger than the one induced by LPS. On the other hand, LPS and dCGN displayed a very different TNF secretion curve in THP-1 cells. These differences suggest that dCGN and LPS could use different activation mechanisms. It is noteworthy that neutralizing antibody to CD14 only partially (<40%) inhibited dCGN-induced TNF secretion (not shown). TLR4 has been recently identified as a surface membrane receptor for CGN in human colonic epithelial cells [41]. Thus, it is possible that TLR4 is activated by dCGN to induce cytokine secretion by monocytes. We can only speculate that TLR4 may have a higher affinity-binding site for dCGN than for LPS, however, this hypothesis remains to be tested.

Another indicator that dCGN stimulate monocytes leading to a more active phenotype is the fact that surface expression of the adhesion molecule ICAM-1 was enhanced in both PBM and THP-1 cells. The over expression of ICAM-1 caused the activated monocytes to form cell aggregates that were more abundant among cells treated with the 40 kDa dCGN. This correlates with the higher expression of ICAM-1 induced by the 40 kDa dCGN, suggesting that the partially degraded CGN is more biologically active. In addition, the cell aggregates are reminiscent of monocyte aggregates, which form multinucleated giant cells (MGC) in patients with Crohn's disease [42]. These giant cells were not observed in healthy individuals or in patients with ulcerative colitis [42]. Moreover, increased expression levels of ICAM-1 and LFA-3 (CD58) were also detected in monocytes from patients with Crohn's disease [43], [44]. Thus, degraded CGN clearly can activate monocytes to express an increased number of ICAM-1 adhesion molecules, therefore being capable of creating the conditions characteristic of Crohn's disease symptomatology, *i.e.* PBM accumulation and MGC formation [45], [46].

The NF-κB pathway regulates genes responsible for ICAM-1 and TNF-α expression. NF-κB activation is associated with the degradation of the inhibitor protein IκB [47]. Indeed, dCGN induced NF-κB activation as shown by degradation of IκBα, translocation of p65 and p50 sub-units to the nucleus and by activation of an NF-κB-responsive luciferase reporter plasmid. Again, a stronger activation of NF-κB was induced by the 40 kDa dCGN compared to 10 kDa dCGN, suggesting that the partially degraded CGN is more biologically active. Our data are in agreement with a previous study showing that native CGN also induced activation of NF-κB in human colonic epithelial cells [48].

NF-κB pathway is often associated to promote cell survival and cancer cell growth, however it has been sometimes reported to behave as a tumor suppressor, arresting cell proliferation [49]. Such relationship between NF-kB activation and cell cycle regulation has been reported in normal human epidermal cells [50]. Indeed, it has been shown that NF-κB activation suppressed cdk4 expression, which is necessary for the transition to S phase. Finally, NF-κB activation was reported to induce growth arrest in normal human keratinocytes by a mechanims involving the cdk inhibitor p21 [51]. The effects of dCGN on p21 and cdk4 expression remain to be studied.

In these studies, we have demonstrated a direct action of dCGN on monocytes. Monocytes exposed to dCGN acquired an inflammatory phenotype that included higher expression of the adhesion molecule ICAM-1 and TNF-α production, *via* the NF-κB pathway. This higher expression of ICAM-1 resulted in formation of cell aggregates similar to those observed in patients with Crohn's disease. We presume that the differential effects of 10 and 40 kDa dCGN to induce these effects on monocytes are tightly linked to their capacity to induce inflammation *in vivo*. However, *in vivo*, macrophages do not come in direct contact with the intestinal lumen and are separated by the epithelial barrier. The way by which dCGN may leave the intestinal lumen and cross the epithelial barrier to reach the macrophages is an intriguing open question. One possible explanation resides in the potential of dCGN to “induce” cellular and paracellular injurious effects at the intestinal epithelial cell monolayer [48].

In conclusion, dCGN inhibited THP-1 cell proliferation *in vitro*, accumulating the cells in the G1 phase of the cell cycle, increased ICAM-1 expression, stimulated ICAM-1-dependent monocyte aggregation *in vitro*, and stimulated TNF-α expression and secretion. These responses were more pronounced following 40 kDa dCGN, and were all linked to NF-κB activation. These results suggest that, although CGN is widely used as a food additive, its degraded forms have an important effect on monocytes characteristic of an inflammatory phenotype.
